# Amino Acid Compositions of 27 Food Fishes and Their Importance in Clinical Nutrition

**DOI:** 10.1155/2014/269797

**Published:** 2014-10-14

**Authors:** Bimal Mohanty, Arabinda Mahanty, Satabdi Ganguly, T. V. Sankar, Kajal Chakraborty, Anandan Rangasamy, Baidyanath Paul, Debajit Sarma, Suseela Mathew, Kurukkan Kunnath Asha, Bijay Behera, Md. Aftabuddin, Dipesh Debnath, P. Vijayagopal, N. Sridhar, M. S. Akhtar, Neetu Sahi, Tandrima Mitra, Sudeshna Banerjee, Prasenjit Paria, Debajeet Das, Pushpita Das, K. K. Vijayan, P. T. Laxmanan, A. P. Sharma

**Affiliations:** ^1^ICAR-Central Inland Fisheries Research Institute, Barrackpore, Kolkata 700120, India; ^2^ICAR-Central Institute of Fisheries Technology, Cochin 682029, India; ^3^ICAR-Central Marine Fisheries Research Institute, Cochin 682018, India; ^4^ICAR-Central Institute of Freshwater Aquaculture, Bhubaneswar 751002, India; ^5^ICAR-Directorate of Coldwater Fisheries Research, Bhimtal, Uttarakhand 263136, India

## Abstract

Proteins and amino acids are important biomolecules which regulate key metabolic pathways and serve as precursors for synthesis of biologically important substances; moreover, amino acids are building blocks of proteins. Fish is an important dietary source of quality animal proteins and amino acids and play important role in human nutrition. In the present investigation, crude protein content and amino acid compositions of important food fishes from different habitats have been studied. Crude protein content was determined by Kjeldahl method and amino acid composition was analyzed by high performance liquid chromatography and information on 27 food fishes was generated. The analysis showed that the cold water species are rich in lysine and aspartic acid, marine fishes in leucine, small indigenous fishes in histidine, and the carps and catfishes in glutamic acid and glycine. The enriched nutrition knowledge base would enhance the utility of fish as a source of quality animal proteins and amino acids and aid in their inclusion in dietary counseling and patient guidance for specific nutritional needs.

## 1. Introduction

Amino acids are important biomolecules that both serve as building blocks of proteins and are intermediates in various metabolic pathways. They serve as precursors for synthesis of a wide range of biologically important substances including nucleotides, peptide hormones, and neurotransmitters. Moreover, amino acids play important roles in cell signaling and act as regulators of gene expression and protein phosphorylation cascade [1], nutrient transport and metabolism in animal cells [2], and innate and cell-mediated immune responses.

Amino acids are mainly obtained from proteins in diet and the quality of dietary protein is assessed from essential to nonessential amino acid ratio. High quality proteins are readily digestible and contain the dietary essential amino acids (EAA) in quantities that correspond to human requirements [3]. Proteins, the most abundant macromolecules found in biological systems, are present in diverse forms such as structural elements, enzymes, hormones, antibodies, receptors, signaling molecules, and so forth, having specific biological functions. Protein is necessary for key body functions including provision of essential amino acids and development and maintenance of muscles. Inadequate uptake of quality proteins and calories in diet leads to protein-energy malnutrition (PEM) (or protein-calorie malnutrition, PCM) which is the most lethal form of malnutrition/hunger. Kwashiorkor and marasmus, the extreme conditions of PCM mostly observed in children, are caused by chronic deficiency of protein and energy, respectively. PCM also occurs in adults who are under chronic nutritional deficiency. About 870 million people in the world are suffering from chronic protein malnutrition; 80% of children suffering from PCM are from developing countries [3, 4]. Fish, in this context, can play a vital role as it is an important and cheaper source of quality animal proteins. Therefore, there is a need to generate and document nutritional information on the numerous varieties and species of food fishes available. In comparison to the other sources of dietary animal proteins, consumers have wide choice for fish as far as affordability is concerned as there are many varieties and species of fishes available, especially in the tropical countries [5]. The present study was undertaken to generate information on protein content and amino acid composition of important food fishes with the objective of enhancing the scope for their utility in clinical nutrition for dietary counseling.

## 2. Materials and Methods

### 2.1. Ethical Statement

The authors confirm that all the research done meets the ethical guidelines, including adherence to the legal requirements of the study country.

### 2.2. Sample Collection and Processing

Freshly caught fishes were collected from either the landing centers or the local fish markets and were brought to the laboratory in ice. A total of 27 species included for amino acid profiling were the carps* Catla catla*,* Labeo rohita*, and* Cirrhinus mrigala*, catfishes* Sperata seenghala*,* Heteropneustes fossilis*, and* Clarias batrachus,* the small indigenous fishes* Amblypharyngodon mola*,* Puntius sophore*,* Anabas testudineus* (all fresh water fishes), and* Tenualosa ilisha* (anadromous), the cold water fishes* Oncorhynchus mykiss*,* Tor putitora*,* Schizothorax richardsonii*,* Neolissochilus hexagonolepis*, and* Cyprinus carpio*; the marine fishes* Thunnus albacares*,* Stolephorus waitei*,* Stolephorus commersonii, Rastrelliger kanagurta*,* Nemipterus japonicas*,* Sardinella longiceps*,* Katsuwonus pelamis*,* Epinephelus *spp.,* Leiognathus splendens*, and* Trichiurus lepturus,* and the shellfishes* Crassostrea madrasensis*,* Perna viridis*. Fishes were cleaned, descaled, degutted, minced, homogenized, and stored at −40°C until used.

### 2.3. Amino Acid Analysis

The crude protein content was determined by Kjeldahl method [6]. Amino acid composition was determined following Ishida et al. [7] and has been described earlier [8]. Briefly, muscle protein was hydrolyzed with 6N hydrochloric acid at 110°C under anaerobic condition for 24 h. The hydrolyzed samples were neutralized with 6N NaOH and were derivatized using a kit (AccQ-Fluor Reagent, WAT052880, Waters). The derivatized samples were injected in high performance liquid chromatography (HPLC) (1525, Waters) equipped with a C_18 _RP column and a fluorescence detector (2475, Waters). The amino acids were identified and quantified by comparing with the retention times and peak areas of standards (WAT088122, Waters). For the tryptophan analysis, minced meat was digested with 5% (w/v) NaOH for 24 h and neutralized to pH 7.0 with 6N HCl. Tryptophan content was measured spectrophotometrically at 530 nm [9]. All data have been presented as mean ± standard deviation.

## 3. Results and Discussion

The physiological role of dietary proteins is to provide substrates required for the synthesis of body proteins and other metabolically important nitrogen-containing compounds. Therefore, the content of the nutritionally indispensable amino acids (AAs) in food proteins is usually the primary determinant of nutritional quality of protein [10]. Moreover, amino acids are associated with health issues and amino acid deficiencies lead to a number of diseases. Hence, knowledge of the amino acid composition of foods serves as a basis for establishing their potential nutritive value. It may also allow evaluation of changes in nutritive value that may arise in the preparation, processing, and storage of foods [11].

AAs have been traditionally classified as nutritionally essential (EAA), “nonessential” (NEAA) or conditionally essential (CEAA) [1]. Arginine, cystine, histidine, leucine, lysine, methionine, threonine, tryptophan, tyrosine, and valine are the EAAs, glutamine, glutamic acid, glycine, proline, and taurine are CEAA, and aspartic acid, serine, and alanine are the NEAA for human nutrition. However, recently the concept of functional amino acids (FAAs) has been proposed. FAAs are those which participate and regulate key metabolic pathways to improve health, survival, growth, development, lactation, and reproduction of the organisms [1, 12]. The FAAs also hold great promise in prevention and treatment of metabolic diseases (e.g., obesity, diabetes, and cardiovascular disorders), intrauterine growth restriction, infertility, intestinal and neurological dysfunction, and infectious disease. Arginine, cystine, leucine, methionine, tryptophan, tyrosine, aspartate, glutamic acid, glycine, proline, and taurine have been classified as FAA in human nutrition [12].

Fish is an important source of quality animal proteins and it has been reported that fish protein has greater satiety effect than other sources of animal proteins like beef and chicken [13]. In comparison to the other sources of dietary animal proteins, consumers have wide choice for fish as far as affordability is concerned as there are many varieties and species of fishes available, especially in the tropical countries [4]. Here, we report the crude protein content and amino acid composition of 27 food fishes from the Indian subcontinent (Tables 1 and 2) which could be useful in patient counseling and recommending species for patients with specific requirements and thus could be useful in clinical medicine. The distributions of amino acid in different species are discussed below. There was no appreciable variation in amino acid composition of fishes of the same species from different locations.

Arginine plays an important role in cell division, wound healing, ammonia removal, immune function, and hormone release. It is also the precursor for biological synthesis of nitric oxide which plays important roles in neurotransmission, blood clotting, and maintenance of blood pressure. It is supplemented for recovery in a number of diseases like sepsis, preeclampsia, hypertension, erectile dysfunction, anxiety, and so forth. Arginine contents of cold water fishes* O. mykiss* (6.5 ± 0.3 g 100 g^−1^ protein),* T. putitora,* and* N. hexagonolepis* were found to be very high among the fishes studied and can be recommended in arginine deficiency [14]. Similar levels of arginine have been reported in the small forage fish capelin (*Mallotus villosus*) (5.70 ± 0.02%) [15].

Leucine is the only dietary amino acid that can stimulate muscle protein synthesis [16] and has important therapeutic role in stress conditions like burn, trauma, and sepsis [17]. As a dietary supplement, leucine has been found to slow the degradation of muscle tissue by increasing the synthesis of muscle proteins. Leucine was very high in marine fishes* S. waitei* and* R. kanagurta* (10.4 ± 0.4 and 10.3 ± 0.4 g 100^−1^ g protein, resp.), carps* L. rohita* and* C. mrigala,* and catfishes* C. batrachus* and* H. fossilis* (Table 1) which is higher than European seabass (7.21 ± 0.56%), gilthead seabream (7.27 ± 0.80%), and turbot (5.91 ± 0.69%) [18].

Methionine is used for treating liver disorders, improving wound healing, and treating depression, alcoholism, allergies, asthma, copper poisoning, radiation side effects, schizophrenia, drug withdrawal, and Parkinson's disease [19]. Methionine content of the marine fish* S. waitei* (4.0 ± 0.4 g 100^−1^ g protein) and cold water fish* T. putitora* (3.6 ± 0.3 g 100^−1^ g protein) was found to be highest among the fishes and is even higher than that found in mutton [20] and comparable to that of murrels* Channa striatus* (3.4 ± 0.11%),* Channa micropeltes* (4.0 ± 0.91%), and* Channa lucius* (3.6 ± 0.16) [21].

Glutamic acid plays an important role in amino acid metabolism because of its role in transamination reactions and is necessary for the synthesis of key molecules, such as glutathione which are required for removal of highly toxic peroxides and the polyglutamate folate cofactors. This amino acid was found to be one of the most abundant amino acids in the carps* C. catla*,* L. rohita*, and* C. mrigala* and catfishes* C. batrachus* and* H. fossilis* (Table 1). Similar values of glutamic acid have been reported in other fish species like mackerel [22] and red salmon [23] and in beef also [24]. Glycine plays an important role in metabolic regulation, preventing tissue injury, enhancing anti-antioxidant activity, promoting protein synthesis and wound healing, and improving immunity and treatment of metabolic disorders in obesity, diabetes, cardiovascular disease, ischemia-reperfusion injuries, cancer, and various inflammatory diseases [2]. The catfish* H. fossilis* was found to contain the highest amount of glycine followed by* A. testudineus* (Table 1) which was much higher than the glycine content of European seabass, gilthead seabream, turbot,* Channa striatus*,* Channa micropeltes*, and* Channa lucius* [18, 21].

Tryptophan is a precursor for serotonin, a brain neurotransmitter theorized to suppress pain. Free tryptophan enters the brain cells to form serotonin. Thus, tryptophan supplementation has been used to increase serotonin production in attempt to increase tolerance to pain [25]. Tryptophan is also the precursor of melatonin, tryptamine, and kynurenine and has an important role in the functioning of neurotransmitters like dopamine and nor-dopamine. Tryptophan supplement is used in treatment of pain, insomnia, depression, seasonal affective disorder, bulimia, premenstrual dysphoric disorder, attention deficit/hyperactivity disorder, and chronic fatigue [26]. The fish* T. putitora* was found to contain the highest amount of tryptophan among the fishes studied (Table 1).

Histidine plays multiple roles in protein interaction [27] and is also a precursor of histamine. It is also needed for growth and repair of tissue, for maintenance of the myelin sheaths, and in removing heavy metals from the body [28]. The marine fish* Rastrelliger kanagurta* was found to have high amount of histidine. The small indigenous fishes* A. testudineus*,* A. mola,* and* P. sophore* [29] were also found to be rich in histidine.

Lysine is an EAA which is extensively required for optimal growth and its deficiency leads to immunodeficiency [30]. Lysine is used for preventing and treating cold sores. It is taken by mouth or applied directly to the skin for this use. Lysine content was very high in* S. commersonii* (16.1 ± 0.9 g 100^−1 ^g protein) and* T. putitora* (9.4 ± 0.6 g 100^−1 ^g protein). The amino acid content of* T. putitora* was similar to that of* Channa striatus* (9.7 ± 0.57%),* Channa micropeltes* (10.9 ± 1.05%), and* Channa lucius* (10.1 ± 1.42%) [21].

Threonine is used for treating various nervous system disorders including spinal spasticity, multiple sclerosis, familial spastic paraparesis, and amyotrophic lateral sclerosis [31]. Threonine content of* S. waitei* was found to be highest among the fish species studied (Table 1). Therefore, this fish can serve as a natural supplement for threonine.

Isoleucine is a branched chain amino acid and is needed for muscle formation and proper growth [32]. Chronic renal failure (CRF) patients on hemodialysis have low plasma level of the branched chain amino acids (BCAA) leucine, isoleucine, and valine. The abnormalities in the plasma amino acid pool can be corrected with appropriate high-protein supplements [33].* O. mykiss* was found to contain the highest amount of isoleucine among the fish species studied (6.5 g 100 g^−1^ protein) followed by* L. rohita* and can be used for isoleucine supplementation.

Although NEAA are synthesized* de novo* in the body, some of the nutritionally NEAA play important roles in regulating gene expression and micro-RNA levels, cell signaling, blood flow, nutrient transport and metabolism in animal cells, development of brown adipose tissue, intestinal microbial growth and metabolism, anti-oxidative responses, and innate and cell-mediated immune responses [1]. Aspartic acid (FAA) is the precursor of AAs methionine, threonine, isoleucine, and lysine and regulates the secretion of important hormones. Similarly, serine is the precursor of glycine, cysteine, and tryptophan and plays many important roles in cell signaling. Serine is also being used for treatment of schizophrenia. Aspartic acid and serine content of* S. commersonii* was found to be highest among the fishes studied, followed by* R. kanagurta*.

The knowledge base enriched with amino acid composition data of 27 important food fishes would be useful in clinical nutrition for issuing patient advisory, dietary guidance, and counseling. Although cooking and boiling cause loss in the content of amino acids to varied degrees [34], the final content is proportional to the crude content [35]. Therefore, in general, the cold water species can be recommended for lysine and aspartic acid, marine fishes for leucine, small indigenous fishes for histidine, and the carps and catfishes for glutamic acid and glycine. However, for specific patient need the amino acid composition data of individual species, as given in Table 1, would be useful.

## Figures and Tables

**(a):** 

Amino acids (g 100 g^−1^ protein)	*Catla catla *	*Labeo rohita *	*Cirrhinus mrigala *	*Sperata seenghala *	*Clarias batrachus *	*Heteropneustes fossilis *	*Anabas testudineus *	*Puntius sophore *	*Amblypharyngodon mola *
	Fresh water fishes								
Essential amino acids (EAAs)									
Arg^c^	1.5 ± 0.4	0.8 ± 0.1	0.9 ± 0.2	nd	2.5 ± 0.5	1.5 ± 0.2	2.1 ± 0.4	0.1 ± 0.03	nd
His	5.3 ± 0.4	3.8 ± 0.5	4.3 ± 0.3	1.1 ± 0.2	3.9 ± 0.2	3.6 ± 0.3	3.9 ± 0.4	1.4 ± 0.3	2.8 ± 0.4
Iso	5.9 ± 0.2	6.2 ± 0.3	5.9 ± 0.4	0.4 ± 0.1	4.7 ± 0.2	5.6 ± 0.3	5.4 ± 0.3	0.2 ± 0.0	0.3 ± 0.0
Leu^c^	7.7 ± 0.2	9.0 ± 0.5	8.4 ± 0.4	0.7 ± 0.1	8.1 ± 0.3	8.2 ± 0.2	8.2 ± 0.3	0.4 ± 0.0	0.4 ± 0.0
Lys	3.6 ± 0.3	2.9 ± 0.5	4.8 ± 0.3	0.3 ± 0.0	4.4 ± 0.3	3.9 ± 0.4	3.0 ± 0.2	nd	
Met^c^	1.9 ± 0.1	1.9 ± 0.1	1.6 ± 0.1	0.14 ± 0.0	2.85 ± 0.3	1.3 ± 0.3	1.6 ± 0.1	0.1 ± 0.0	0.1 ± 0.0
Phe	4.8 ± 0.2	5.5 ± 0.2	6.1 ± 0.3	0.4 ± 0.1	3.7 ± 0.3	6.2 ± 0.1	6.3 ± 0.2	nd	0.3 ± 0.0
Thr	5.5 ± 0.3	5.7 ± 0.6	5.8 ± 0.7	0.6 ± 0.1	4.6 ± 0.1	5.9 ± 0.3	5.5 ± 0.3	0.3 ± 0.0	0.4 ± 0.0
Tyr^c^	1.2 ± 0.3	1.3 ± 0.2	0.9 ± 0.1	nd	0.9 ± 0.2	0.9 ± 0.1	1.0 ± 0.0	nd	nd
Val	6.7 ± 1.1	6.0 ± 0.9	7.2 ± 1.2	0.4 ± 0.0	5.7 ± 1.1	5.8 ± 1.0	7.3 ± 1.3	0.2 ± 0.0	0.3 ± 0.0
Trp^c^	1.0 ± 0.1	0.5 ± 0.0	0.6 ± 0.0	0.2 ± 0.0	1.1 ± 0.0	0.6 ± 0.0	1.4 ± 0.3	0.1 ± 0.0	0.2 ± 0.0
Cys^c^	0.1 ± 0.0	0.1 ± 0.0	0.1 ± 0.0	nd	0.2 ± 0.0	0.1 ± 0.0	0.2 ± 0.0	nd	nd
Glu^ac^	13.8 ± 3.5	14.6 ± 3.9	14.8 ± 4.0	1.6 ± 0.3	14.5 ± 3.6	16.0 ± 4.2	13.1 ± 4.0	0.7 ± 0.1	1.0 ± 0.2
Gly^ac^	13.7 ± 3.4	13.6 ± 3.6	13.4 ± 3.2	0.4 ± 0.0	13.6 ± 3.1	15.4 ± 3.6	15.3 ± 4.1	0.6 ± 0.1	0.5 ± 0.1
Pro^ac^	0.4 ± 0.0	0.5 ± 0.1	0.9 ± 0.1	0.6 ± 0.1	1.3 ± 0.3	0.9 ± 0.1	1.6 ± 0.1	0.6 ± 0.1	0.6 ± 0.1
Nonessential amino acids (NEAAs)									
Ala	7.2 ± 1.2	7.8 ± 1.1	5.9 ± 0.8	0.3 ± 0.0	6.4 ± 1.1	5.6 ± 1.2	7.3 ± 1.5	0.2 ± 0.0	0.2 ± 0.0
Asp^c^	10.4 ± 2.3	10.0 ± 2.5	10.9 ± 2.0	2.3 ± 0.9	11.1 ± 3.2	12.3 ± 3.5	10.8 ± 2.5	1.2 ± 0.2	1.4 ± 0.1
Ser	5.9 ± 1.5	6.3 ± 1.9	6.3 ± 1.5	0.4 ± 0.0	6.1 ± 2.3	6.5 ± 2.4	5.1 ± 1.6	0.2 ± 0.0	0.3 ± 0.0

Crude protein (%)	16.2 ± 0.5	15.9 ± 0.4	15.5 ± 0.5	19.0 ± 1.3	16.4 ± 0.3	16.3 ± 0.4	16.9 ± 0.5	16.3 ± 0.8	16.3 ± 0.4

**(b):** 

Amino acids (g 100 g^−1^ protein)	*Tor putitora *	*Neolissochilus hexagonolepis *	*Oncorhynchus mykiss *	*Schizothorax richardsonii *	*Cyprinus carpio *	*Tenualosa ilisha *	*Sardinella longiceps *	*Nemipterus japonicus *	*Thunnus albacares *
	Cold water fishes					Anadromous	Marine fishes		
Essential amino acids (EAA)									
Arg^c^	4.2 ± 0.2	2.1 ± 0.4	6.5 ± 0.3	2.3 ± 0.3	2.1 ± 0.3	nd	0.6 ± 0.2	1.1 ± 0.3	0.3 ± 0.1
His	0.5 ± 0.0	0.9 ± 0.1	1.1 ± 0.1	0.6 ± 0.0	0.4 ± 0.0	1.7 ± 0.3	0.4 ± 0.0	2.8 ± 0.4	7.7 ± 0.5
Iso	3.7 ± 0.4	1.1 ± 0.3	6.5 ± 0.4	1.3 ± 0.2	0.8 ± 0.1	0.5 ± 0.1	0.4 ± 0.0	5.1 ± 0.2	4.9 ± 0.3
Leu^c^	7.6 ± 0.3	2.1 ± 0.2	5.7 ± 0.2	2.3 ± 0.1	1.6 ± 0.1	0.7 ± 0.1	0.6 ± 0.0	10.1 ± 0.2	9.1 ± 0.3
Lys	9.4 ± 0.6	2.0 ± 0.2	3.5 ± 0.3	2.3 ± 0.4	1.1 ± 0.3	0.3 ± 0.0	0.7 ± 0.1	2.8 ± 0.3	12.4 ± 0.9
Met^c^	3.6 ± 0.3	1.1 ± 0.1	3.1 ± 0.2	1.2 ± 0.1	0.8 ± 0.1	0.1 ± 0.0	0.3 ± 0.3	2.4 ± 0.2	2.7 ± 0.3
Phe	5.5 ± 0.3	0.9 ± 0.1	3.0 ± 0.1	0.9 ± 0.1	0.7 ± 0.1	0.4 ± 0.1	0.6 ± 0.1	3.7 ± 0.3	3.9 ± 0.3
Thr	3.9 ± 0.2	0.9 ± 0.2	5.9 ± 0.5	0.9 ± 0.1	0.9 ± 0.3	0.6 ± 0.1	4.1 ± 0.5	6.4 ± 0.7	6.6 ± 0.8
Tyr^c^	5.7 ± 0.5	0.9 ± 0.1	8.4 ± 0.8	nd	0.8 ± 0.2	nd	0.4 ± 0.0	1.3 ± 0.3	1.5 ± 0.3
Val	3.8 ± 0.9	1.6 ± 0.3	4.9 ± 0.8	1.7 ± 0.5	1.3 ± 0.4	0.4 ± 0.0	4.4 ± 0.9	8.6 ± 1.3	8.2 ± 0.4
Trp^c^	6.5 ± 0.9	0.4 ± 0.0	6.2 ± 1.0	0.4 ± 0.0	0.9 ± 0.0	0.2 ± 0.0	nd	2.3 ± 0.7	1.6 ± 0.1
Cys^c^	nd	0.2 ± 0.0	nd	0.2 ± 0.0	0.04 ± 0.0	nd	0.2 ± 0.0	0.4 ± 0.1	nd
Glu^ac^	9.6 ± 1.3	4.5 ± 1.2	4.9 ± 1.2	4.2 ± 1.0	4.2 ± 1.1	1.2 ± 0.3	1.1 ± 0.4	16.55 ± 1.2	11.1 ± 3.5
Gly^ac^	7.5 ± 1.5	2.3 ± 0.4	6.9 ± 1.2	2.3 ± 0.9	3.2 ± 0.9	0.5 ± 0.0	0.4 ± 0.0	7.6 ± 1.5	8.5 ± 2.2
Pro^ac^	6.7 ± 2.1	1.1 ± 0.2	9.6 ± 1.4	1.3 ± 0.3	1.2 ± 0.3	0.5 ± 0.0	0.4 ± 0.0	1.1 ± 0.3	0.9 ± 0.0
Nonessential amino acids (NEAA)									
Ala	5.1 ± 0.9	4.1 ± 1.3	6.1 ± 1.1	4.0 ± 0.8	3.7 ± 0.7	0.3 ± 0.1	0.4 ± 0.0	8.1 ± 1.9	6.1 ± 1.1
Asp	7.6 ± 1.8	7.1 ± 2.8	8.5 ± 1.9	5.2 ± 1.3	6.2 ± 1.9	1.8 ± 0.6	nd	11.1 ± 2.3	8.5 ± 1.9
Ser	4.1 ± 1.3	4.9 ± 1.3	6.6 ± 2.4	5.5 ± 2.1	5.5 ± 2.1	0.5 ± 0.0	5.5 ± 0.0	6.8 ± 2.3	6.6 ± 2.4

Crude protein (%)	17.0 ± 0.2	18.2 ± 0.3	17.2 ± 0.0	16.3 ± 0.1	17.2 ± 0.2	20.7 ± 2.7	17.1 ± 1.4	15.4 ± 0.2	23.9 ± 0.1

**(c):** 

Amino acids (g 100 g^−1^ protein)	*Stolephorus waitei *	*Stolephorus commersonii *	*Rastrelliger kanagurta *	*Katsuwonus pelamis *	*Epinephelus spp *	*Leiognathus splendens *	*Trichiurus lepturus *	*Crassostrea madrasensis *	*Perna viridis *
	Marine fishes							Bivalve molluscs	
Essential amino acids (EAA)									
Arg^c^	0.8 ± 0.1	nd	0.7 ± 0.2	1.0 ± 0.1	0.9 ± 0.2	nd	1.0 ± 0.2	2.7 ± 0.3	0.2 ± 0.0
His	4.4 ± 0.4	4.2 ± 0.3	7.9 ± 0.6	0.5 ± 0.0	0.5 ± 0.0	nd	0.6 ± 0.1	0.4 ± 0.1	0.03 ± 0.0
Iso	4.2 ± 0.5	5.2 ± 0.2	4.7 ± 0.3	0.5 ± 0.0	0.4 ± 0.0	0.3 ± 0.0	0.5 ± 0.0	0.5 ± 0.1	0.05 ± 0.0
Leu^c^	10.4 ± 0.4	7.9 ± 0.5	10.3 ± 0.4	0.9 ± 0.1	0.7 ± 0.0	0.7 ± 0.0	0.9 ± 0.1	1.3 ± 0.2	0.1 ± 0.0
Lys	5.7 ± 0.2	16.1 ± 0.9	4.6 ± 0.6	1.2 ± 0.1	0.9 ± 0.1	0.9 ± 0.0	0.9 ± 0.0	1.1 ± 0.2	0.09 ± 0.0
Met^c^	4.0 ± 0.4	1.0 ± 0.2	3.4 ± 0.1	0.4 ± 0.0	0.4 ± 0.0	0.4 ± 0.1	0.5 ± 0.0	0.4 ± 0.1	0.02 ± 0.0
Phe	4.3 ± 0.2	3.6 ± 0.1	4.2 ± 0.2	0.5 ± 0.2	0.6 ± 0.0	0.6 ± 0.0	0.6 ± 0.0	0.7 ± 0.1	0.06 ± 0.0
Thr	7.9 ± 0.9	5.4 ± 0.8	7.1 ± 0.9	0.6 ± 0.0	0.6 ± 0.0	0.5 ± 0.0		0.7 ± 0.0	0.6 ± 0.1
Tyr^c^	1.1 ± 0.1	0.2 ± 0.0	1.2 ± 0.2	0.3 ± 0.1	1.4 ± 0.1	nd	0.5 ± 0.0	0.4 ± 0.0	0.03 ± 0.0
Val	5.6 ± 0.9	5.6 ± 0.5	7.1 ± 1.3	0.6 ± 0.1	0.5 ± 0.1	0.5 ± 0.0	0.6 ± 0.1	0.6 ± 0.1	0.05 ± 0.0
Trp^c^	2.1 ± 0.5	2.1 ± 0.6	1.2 ± 0.3	nd	nd	nd	nd	nd	nd
Cys^c^	nd	nd	nd	0.6 ± 0.1	0.6 ± 0.1	0.5 ± 0.1		0.2 ± 0.0	0.03 ± 0.0
Glu^ac^	13.0 ± 3.9	13.6 ± 4.2	13.9 ± 4.0	2.1 ± 0.3	1.6 ± 0.6	nd	nd	2.5 ± 0.9	0.2 ± 0.0
Gly^ac^	9.8 ± 2.3	8.8 ± 1.5	8.3 ± 1.3	0.4 ± 0.0	0.4 ± 0.1	nd	nd	1.9 ± 0.5	0.1 ± 0.0
Pro^ac^	1.8 ± 0.2	1.5 ± 0.3	1.5 ± 0.2	0.5 ± 0.0	0.5 ± 0.0	nd	nd	0.7 ± 0.1	0.07 ± 0.0
Nonessential amino acids (NEAA)									
Ala	4.1 ± 1.1	6.4 ± 1.2	4.8 ± 1.1	0.6 ± 0.1	0.5 ± 0.0	0.6 ± 0.0	nd	1.2 ± 0.2	0.08 ± 0.0
Asp	9.9 ± 3.0	12.3 ± 3.6	10.1 ± 3.2	nd	nd	nd	nd	nd	0.1 ± 0.0
Ser	7.2 ± 2.9	6.0 ± 2.4	6.9 ± 12.1	0.4 ± 0.0	0.6 ± 0.1	nd	nd	1.2 ± 0.3	0.09 ± 0.0

Crude protein (%)	20.3 ± 0.1	16.4 ± 0.1	19.2 ± 0.1	22.4 ± 2.9	18.1 ± 1.1	17.2 ± 1.6	17.9 ± 1.5	16.8 ± 0.1	11.0 ± 0.1

Classification of AA as nutritionally “essential” or “nonessential” or “conditionally essential” is as per Wu 2013 [12].

^a^Conditionally essential amino acids; ^c^functional amino acids as per human nutrition (Wu 2010, 2013) [1, 12].

Values are reported as mean ± standard deviation of three replicates; nd: not detected.

**Table 2:** Fish species rich in different amino acids. Fish species rich in specific amino acids among the species studied are listed (ref. Table 1).

Amino acids	Species rich in particular amino acid
Essential amino acids	
Arg^c^	*Oncorhynchus mykiss, Tor putitora, Neolissochilus hexagonolepis *
His	*Rastrelliger kanagurta, Catla catla*, *Stolephorus waitei*, *Amblypharyngodon mola, Puntius sophore *
Iso	*Oncorhynchus mykiss, Labeo rohita, Stolephorus commersonii *
Leu^c^	*Stolephorus waitei, Rastrelliger kanagurta*, *Labeo rohita *
Lys	*Stolephorus commersonii, Thunnus albacores, Tor putitora *
Met^c^	*Stolephorus waitei, Tor putitora, Rastrelliger kanagurta *
Phe	*Cirrhinus mrigala, Catla catla, Labeo rohita *
Thr	*Thunnus albacores, Nemipterus japonicus, Stolephorus waitei, Stolephorus commersonii *
Tyr^c^	*Oncorhynchus mykiss, Tor putitora *
Val	*Nemipterus japonicas, Cirrhinus mrigala, Rastrelliger kanagurta *
Trp^c^	*Tor putitora *
Glu^ac^	*Cirrhinus mrigala, Catla catla, Labeo rohita *
Gly^ac^	*Cirrhinus mrigala, Catla catla, Labeo rohita *
Pro^ac^	*Oncorhynchus mykiss, Tor putitora *
Nonessential amino acids	
Ala	*Nemipterus japonicus*, *Labeo rohita, Catla catla *
Asp	*Stolephorus commersonii, Heteropneustes fossilis, Clarias batrachus *
Ser	*Stolephorus commersonii, Nemipterus japonicas, Thunnus albacares *

^a^Conditionally essential amino acids; ^c^functional amino acids as per human nutrition.
